# CATMA, a comprehensive genome-scale resource for silencing and transcript profiling of Arabidopsis genes

**DOI:** 10.1186/1471-2105-8-400

**Published:** 2007-10-18

**Authors:** Gert Sclep, Joke Allemeersch, Robin Liechti, Björn De Meyer, Jim Beynon, Rishikesh Bhalerao, Yves Moreau, Wilfried Nietfeld, Jean-Pierre Renou, Philippe Reymond, Martin TR Kuiper, Pierre Hilson

**Affiliations:** 1 Department of Plant Systems Biology, VIB, Technologiepark 927, 9052 Ghent, Belgium; 2 Department of Molecular Genetics, Ghent University, 9052 Ghent, Belgium; 3 ESAT/SISTA, K.U.Leuven, Kasteelpark Arenberg 10, 3001 Heverlee, Belgium; 4 Swiss Institute of Bioinformatics, Genopode building, 1015 Lausanne, Switzerland; 5 Department of Plant Genetics & Biology, HRI, University of Warwick, Warwick, CV35 9EF, UK; 6 Department of Forest Genetics and Plant Physiology, SLU, SE-901 83 Umeå, Sweden; 7 Department of Vertebrate Genomics, Max Planck Institute for Molecular Genetics, 14195 Berlin, Germany; 8 Unité de Recherche en Génomique Végétale, INRA, 91057 Evry cedex, France; 9 Department of Plant Molecular Biology, Biophore Building, University of Lausanne, 1015 Lausanne, Switzerland; 10 Genomics Platform, Parco Tecnologico Padano, Via Einstein, 26900 Lodi, Italy; 11 Microarray facility, VIB, Gasthuisberg Onderwijs en Navorsing 1, Herestraat 49, 3000 Leuven, Belgium

## Abstract

**Background:**

The Complete Arabidopsis Transcript MicroArray (CATMA) initiative combines the efforts of laboratories in eight European countries [1] to deliver gene-specific sequence tags (GSTs) for the Arabidopsis research community. The CATMA initiative offers the power and flexibility to regularly update the GST collection according to evolving knowledge about the gene repertoire. These GST amplicons can easily be reamplified and shared, subsets can be picked at will to print dedicated arrays, and the GSTs can be cloned and used for other functional studies. This ongoing initiative has already produced approximately 24,000 GSTs that have been made publicly available for spotted microarray printing and RNA interference.

**Results:**

GSTs from the CATMA version 2 repertoire (CATMAv2, created in 2002) were mapped onto the gene models from two independent Arabidopsis nuclear genome annotation efforts, TIGR5 and PSB-EuGène, to consolidate a list of genes that were targeted by previously designed CATMA tags. A total of 9,027 gene models were not tagged by any amplified CATMAv2 GST, and 2,533 amplified GSTs were no longer predicted to tag an updated gene model. To validate the efficacy of GST mapping criteria and design rules, the predicted and experimentally observed hybridization characteristics associated to GST features were correlated in transcript profiling datasets obtained with the CATMAv2 microarray, confirming the reliability of this platform. To complete the CATMA repertoire, all 9,027 gene models for which no GST had yet been designed were processed with an adjusted version of the Specific Primer and Amplicon Design Software (SPADS). A total of 5,756 novel GSTs were designed and amplified by PCR from genomic DNA. Together with the pre-existing GST collection, this new addition constitutes the CATMAv3 repertoire. It comprises 30,343 unique amplified sequences that tag 24,202 and 23,009 protein-encoding nuclear gene models in the TAIR6 and EuGène genome annotations, respectively. To cover the remaining untagged genes, we identified 543 additional GSTs using less stringent design criteria and designed 990 sequence tags matching multiple members of gene families (Gene Family Tags or GFTs) to cover any remaining untagged genes. These latter 1,533 features constitute the CATMAv4 addition.

**Conclusion:**

To update the CATMA GST repertoire, we designed 7,289 additional sequence tags, bringing the total number of tagged TAIR6-annotated Arabidopsis nuclear protein-coding genes to 26,173. This resource is used both for the production of spotted microarrays and the large-scale cloning of hairpin RNA silencing vectors. All information about the resulting updated CATMA repertoire is available through the CATMA database http://www.catma.org.

## Background

The Complete Arabidopsis Transcriptome Microarray (CATMA) consortium [1] was created in 2000 to take advantage of the available Arabidopsis genome sequence to enable novel functional genomics approaches. Eight European plant genomics research groups teamed up to produce a comprehensive set of Gene-specific Sequence Tags (GSTs) originally designed for microarray transcript profiling. These GSTs were 150–500 base pairs in length and were selected to have no significant similarity with any other sequence in the genome [2]. The Specific Primer and Amplicon Design Software (SPADS) was written to automate the design of these tags [3]. The resulting GST amplicons can be used as features on spotted microarrays for transcript profiling experiments. Indeed, such CATMA arrays performed as well as, if not better than, the Affymetrix (ATH1) and Agilent (Arabidopsis oligo 2) platforms in terms of specificity, sensitivity and gene coverage [4]. As an academic initiative, CATMA provide the research community with an independent and flexible alternative to commercial arrays. Furthermore, the GSTs can be utilized for posttranscriptional gene silencing when cloned into hairpin RNA expression vectors [5]. The AGRIKOLA consortium has converted the CATMA GST repertoire into hairpin RNA expression vectors [6] and over 2,000 such silencing constructs have been transformed into Arabidopsis to produce knock-down lines [7].

Here, we describe a major effort to create a comprehensive DNA tag repertoire effectively targeting nearly all protein-encoding genes in Arabidopsis. The pre-existing CATMAv2 repertoire was first mapped to recent Arabidopsis genome sequence annotations, TIGR release 5 (TIGR5) (January 2004, [8,9]) and EuGène 040917 (Additional File 1), with the aim of identifying a GST of the highest possible quality for each documented protein-encoding gene model. EuGène results were taken into account because annotation projects focusing on the genome of various species [10,11] have confirmed the quality of the EuGène annotation algorithm [12] and no single algorithm can be perfectly accurate. We implemented the improved and alternative algorithms for the design of GSTs of most of the remaining 'orphan' Arabidopsis protein-encoding genes.

## Results and discussion

### Mapping of CATMA GSTs and gene classification

**Figure 1:**
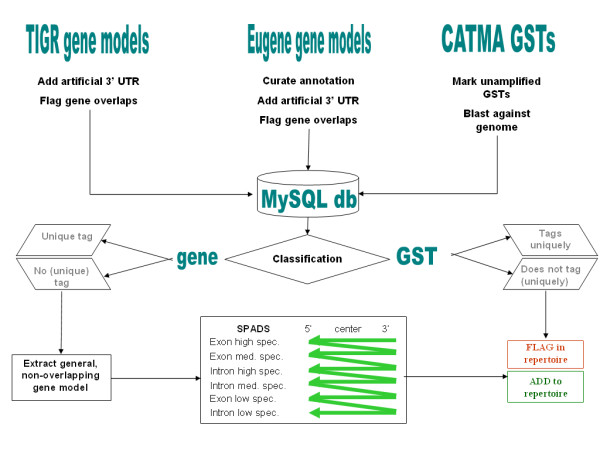
**Overview of the GST classification and design process yielding the CATMAv3 repertoire**. The design and classification process was started with the creation of a MySQL database containing three types of information: the exon coordinates of the TIGR5 annotated protein-coding nuclear genes, the exon coordinates of Eugène 040917, an in-house generated and curated annotation, and the BLAST hit coordinates of the CATMAv2 GSTs, blasted against the Arabidopsis genome. For each annotation source, regions of overlapping genes were marked and gene models that ended with the ORF stop codon were extended with an 'artificial 3' UTR of 150 bp. Information on the prior CATMAv2 GST amplification success or failure was also added to the database. In a second step, both GSTs and genes were classified into five different categories. The classification routine is depicted in Additional File 2 and the categories themselves are described in detail in Table 2. Only successfully amplified GSTs were taken into consideration for the gene classification. When a gene was classified as GE5, it was considered as having a 'unique' tag. When a GST was classified as GST5, it was considered as 'tagging uniquely'. The GST classification was added to the CATMA database, flagging the non-tagging GSTs without actually removing them from the repository. The gene classification was used as a basis for the third and final step, the design of new GSTs for all genes not classified as GE5. To this end, we used the SPADS 1.1.5 software on virtual gene models from which all overlapping exon regions and all exon regions not common to all of the gene's alternative splice forms were removed When no GST can be designed in the most divergent exon regions, SPADS increasingly incorporates less divergent exon regions in its search space (producing GSTs with progressively lower specificity (high, medium or low) and at one point also allows the design of intron-spanning GSTs. At each design level, SPADS scans the gene model from the 3' end to the 5' end. Newly designed GSTs were added to the CATMA database.

To keep up with evolving and increasingly more accurate genome annotations, continued efforts are needed to synchronize probe repertoires with changes in the list of annotated genes. The first CATMA GST design rounds [3,5] were based on earlier Arabidopsis genome annotation releases, namely EuGène 2003, TIGR3 and TIGR4, that were outdated at the time the present work was initiated. Therefore, we first determined which genes described in the more recent EuGène 040917 and TIGR5 (January 2004) annotation releases were still unambiguously tagged by pre-existing GSTs to identify the list of 'orphan' genes that should be considered for upgrading and expanding the GST repertoire (Figure 1). We finally mapped all newly designed GSTs onto the TAIR6 (October 2005) gene models. This work is part of our ongoing efforts to assure the comprehensive nature of the CATMA resources (see also 'Note added in proof').

The first step of the procedure was to position the coordinates of the amplified CATMAv2 GSTs with respect to both the TIGR5 and EuGène 040917 gene models. Briefly, a gene was considered 'tagged' when it contained one or more exonic regions that sufficiently overlapped with at least one GST. A gene was 'uniquely tagged' when this GST had no significant overlap with any other gene. The following criteria were applied to classify a gene as being 'uniquely tagged' by a GST or, vice versa, to classify a GST as 'uniquely tagging' a gene:

a) The gene overlaps with the primary BLAST hit of the GSTs so that the percentage sequence identity of the GST with the corresponding gene region must be at least 99%.

b) At least 100 bp of this matching gene region must be inside an exon.

c) At most 30 bp of the whole GST sequence might overlap with an exonic region of another gene.

d) The GST must not have a significant secondary BLAST hit, i.e. the percentage sequence identity of the GST with any exon of any other gene should be lower than 70%.

Five non-overlapping classes of genes (GE1 to GE5) and GSTs (GST1 to GST5) were defined based on these rules (for more details, see Table 1). Class GE5 and GST5 contain the uniquely tagged genes and the uniquely tagging GSTs, respectively. The flowchart for the classification procedure is presented in Additional File 2.

**Table 1:** Classes of genes and GSTs and class description

Class code	Class name	Class description
**GST classification**		
**GST5**	Uniquely tagging	The GST primary BLAST hit overlaps with exon(s) of an annotated gene over at least 100 bp and none of the GSTs BLAST hits significantly overlap with exon(s) of another gene.
**GST4**	Dubiously tagging	The GST primary BLAST hit overlaps with exon(s) of an annotated gene over at least 100 bp, the primary BLAST hit doesn't share 30 bp with exon(s) of another gene, but the GST has a secondary BLAST hit having more than 70% sequence identity with exon(s) of another gene.
**GST3**	Co- tagging	The GST primary BLAST hit overlaps with exon(s) of an annotated gene over at least 100 bp, but the primary BLAST hit also shares at least 30 bp with exon(s) of another gene.
**GST2**	Insufficiently tagging	The GST primary BLAST hit overlaps with exon(s) of an annotated gene, but over less than 100 bp.
**GST1**	Not tagging	The GST primary BLAST hit overlaps with no annotated exon.
**Gene classification**		
**GE5**	Uniquely tagged	At least one GST tags this gene uniquely, according to the definition of GST5.
**GE4**	Dubiously tagged	At least one GST tags this gene dubiously, according to the definition of GST4. No GST tags this gene uniquely.
**GE3**	Co-tagged	At least one GST co-tags this gene, according to the definition of GST3. No GST tags this gene dubiously or uniquely.
**GE2**	Insufficiently tagged	At least one GST tags this gene insufficiently, according to the definition of GST2. No GST co-tags this gene, nor tags this gene dubiously or uniquely.
**GE1**	Not tagged	The primary BLAST hit of no single GST overlaps with exon(s) of this gene.

The mapping of the CATMAv2 GSTs onto the 26,207 TIGR5 protein-encoding gene models and the subsequent classification resulted in 19,003 (72.5%) genes classified as GE5. Mapping onto the 27,977 EuGène models resulted in 18,193 (65.0%) GE5 genes. The class distribution statistics, including mapping against the TAIR6 (November 2005, [13]) genome annotation release, is detailed in Table 2. The GST/gene correspondence (TAIR6 and EuGène 040917) is listed in Additional File 3.

**Table 2:** Classification of genes and GSTs covered by the CATMAv2 repertoire

**Gene class**	TIGR5 # (%)	TAIR6 # (%)	EuGène # (%)	**GST class**	TIGR5 and EuGène # (%)	TAIR6 and EuGène # (%)
**GE1**	5,674 (21.7%)	5,920 (22.3%)	7,794 (27.8%)	**GST1**	1,231 (5.1%)	1,204 (5.0%)
**GE2**	314 (1.2%)	302 (1.1%)	551 (2.0%)	**GST2**	368 (1.5%)	363 (1.5%)
**GE3**	789 (3,0%)	815 (3%)	994 (3,5%)	**GST3**	315 (1,3%)	317 (1,3%)
**GE4**	427 (1.6%)	428 (1.6%)	445 (1.6%)	**GST4**	750 (3.1%)	752 (3.1%)
**GE5**	19,003 (72.5%)	19,076 (71.9%)	18,193 (65.0%)	**GST5**	21,287 (88.9%)	21,315 (90.0%)
**TOTAL**	26,207	26,541	27,977		23,951	23,951

The numbers refer either to protein-encoding genes of the nuclear genome (Gene class) or to CATMAv2 GSTs that were successfully amplified (GST class).

### SPADS algorithm adjustments

The GST design procedure presented below was based on the previously described SPADS algorithm [3]. The successive steps encoded in SPADS (represented at the bottom of Figure 1) are described in Methods. The script of SPADS 1.1.4 was debugged and optimized, in particular with regard to (i) the verification of uniqueness of PCR primer binding sites and (ii) the program's performance in identifying novel GSTs. The adjusted SPADS version was numbered 1.1.5.

To obtain a quantitative measure of the performance increase between the SPADS versions, we compared their efficiency on the same set of 'orphan' gene models, consisting of 1,823 TIGR5 protein-encoding genes located on chromosome 1 and untagged by the CATMAv2 repertoire. The efficiency of GST design was improved from 57% to 69%, for SPADS 1.1.4 and 1.1.5, respectively. The increased efficiency was at the cost of a slightly reduced specificity. For details, see 'Characteristics of the CATMAv3 addition'.

### Design of the CATMAv3 addition

To design novel GSTs for all the genes not yet uniquely tagged in the CATMAv2 repertoire (classes GE1 to GE4 in Table 2), we first produced an optimized target gene set. This 'orphan' set totaled 9,027 gene models, consisting of 7,204 TIGR5 sequences and 1,823 models predicted additionally by EuGène 040917 that shared no overlap with any genic region in TIGR5 (same or opposite strand). The orphan gene models were slightly modified prior to SPADS processing: (1) as in previous GST design efforts [5], gene models that ended with the ORF stop codon were extended by 150 bp, as a conservative prediction for the presence of a 3' UTR; this extension is henceforth referred to as the 'artificial 3' UTR' (extensions were added to 3,639 TIGR5 and 1,735 EuGène models); (2) regions shared by two overlapping genes were removed (applied to 1,274 TIGR5 and 95 EuGène models) to avoid GSTs tagging of more than one gene; (3) in the case of splice variants, the gene model presented to SPADS was trimmed to contain the exons present in all variants (only concerning TIGR5 models, of which 431 targets contained splice variants).

With SPADS 1.1.5, a GST was successfully designed for 5,756 out of 9,027 target genes (64%). Table 3 presents a breakdown of results according to the four gene classes (GE1-GE4) requiring the design of novel tags. The new set of GSTs, combined with the CATMAv2 repertoire, constituted CATMAv3. Their correspondence with gene models (TAIR6 and EuGène 040917) is listed in Additional File 3. As a first step toward their use in microarray transcript profiling and gene silencing experiments, the tags of this new set were amplified by PCR as described [5] from Arabidopsis genomic DNA, with SPADS-selected primer pairs. The size of all PCR products was analyzed by DNA gel electrophoresis. In total, 5,388 GSTs (93.6%) were detected as products with the predicted size. These results are comparable to the two previous synthesis campaigns. The cloning of these amplified GSTs is described elsewhere [7]. All newly designed tags are listed in Additional File 4. This information is also available on line via the CATMA database [14].

**Table 3:** GST design results of the CATMAv3 addition

Gene class	Annotation	Gene number	Success number	Success rate (%)
GE1	TIGR5	5,674	3,632	64.0
GE1	EuGène 040917	1,733	824	47.4
GE2	TIGR5	314	279	88.9
GE2	EuGène 040917	42	31	73.8
GE3	TIGR5	789	669	85.0
GE3	EuGène 040917	22	9	40.9
GE4	TIGR5	427	304	71.2
GE4	EuGène 040917	26	8	30.8

TOTAL	TIGR5	7,204	4,884	67.8
	EuGène 040917	1,823	872	47.8

### Characteristics of the CATMAv3 addition

**Figure 2:**
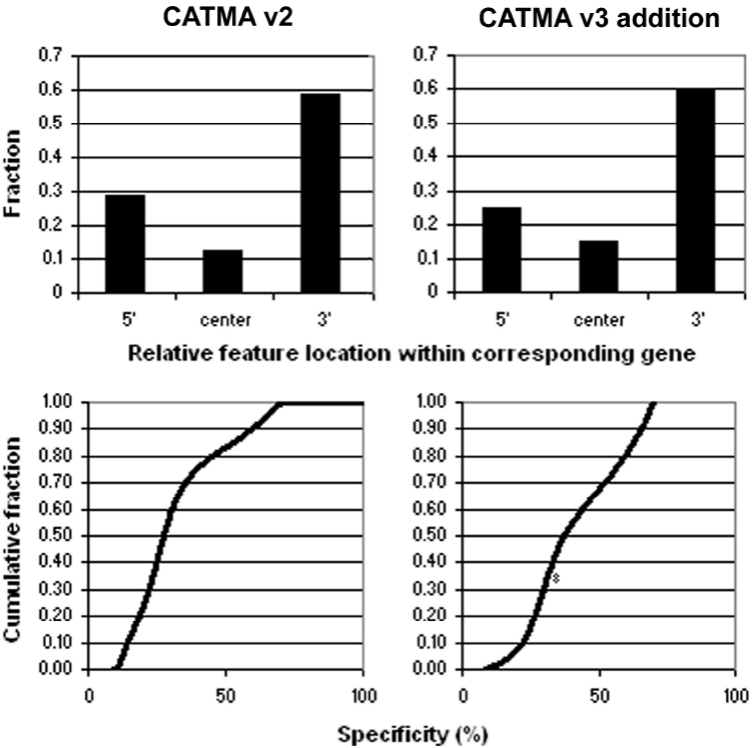
**Comparative analysis of the CATMAv2 repertoire and the CATMAv3 addition**. Quality comparison between the CATMAv2 repertoire (left) and the CATMAv3 repertoire (right). The top and bottom panels show the distribution of the probes with regard to their mapping location on the cognate gene and the cumulative distribution of the probe specificity, measured as the percentage sequence identity of the best non-trivial BLAST hit when comparing the probe against the TIGR5 genome, respectively.

Two parameters were analyzed to investigate whether the new GST set (CATMAv3 addition) has properties similar to the previously designed tags (CATMAv2) and to assess the consistency of the subsequent GST batches: (1) the relative position of the tag within the gene (Figure 2, top), with the 3' location being preferable to 5', because transcript labeling techniques often include oligo-dT priming steps; (2) the distribution of GST specificity measured as the percentage sequence identity of a tag with the next best non-trivial genome BLAST hit (Figure 2, bottom), indicating the likelihood that GST microarray features might yield unspecific hybridization. The comparative analysis indicated that both sets are similar with regard to the relative GST position. The CATMAv3 addition did not show an overall decrease in specificity. However, from Figure 2, a larger fraction of the next best non-trivial BLAST hits has a homology closer to 70% (the absolute overall cut-off) in v3 than in v2. Although it is reasonable to assume that the performance of the majority of the added features would be similar to that of the CATMAv2 repertoire in microarray experiments [4], we examined more closely the cross-hybridization potential for all GSTs. For this purpose, we calculated the melting temperature of the DNA heteroduplex between each GST and their best non-target BLAST hit sequence with the Baldino formula [15], in commonly adopted CATMA microarray hybridization conditions (4×SSC, 50% formamide, 45°C). Conservatively, a GST was flagged for potential cross-hybridization when this predicted melting temperature was equal to or higher than 45°C. This information is available via the CATMA database [14]. The percentages of flagged GSTs were 3.2%, and 9.7% for CATMAv2 and CATMAv3, respectively.

### Experimental validation of GST classification

We reasoned that hybridization signal values would be differently distributed for GST features of different classes (Tables 1 and 2), reflecting their efficacy in detecting expressed sequences. Compared to the best feature class (GST5, tagging a unique gene with sufficient coverage), sequences similar to multiple genes (GST3 and GST4) might hybridize to more than one transcript and, thus display on average a higher signal. Inversely, features with insufficient gene coverage (GST2) or no discernable gene model (GST1) would have a lower signal, with GST1 features theoretically reporting background.

**Figure 3:**
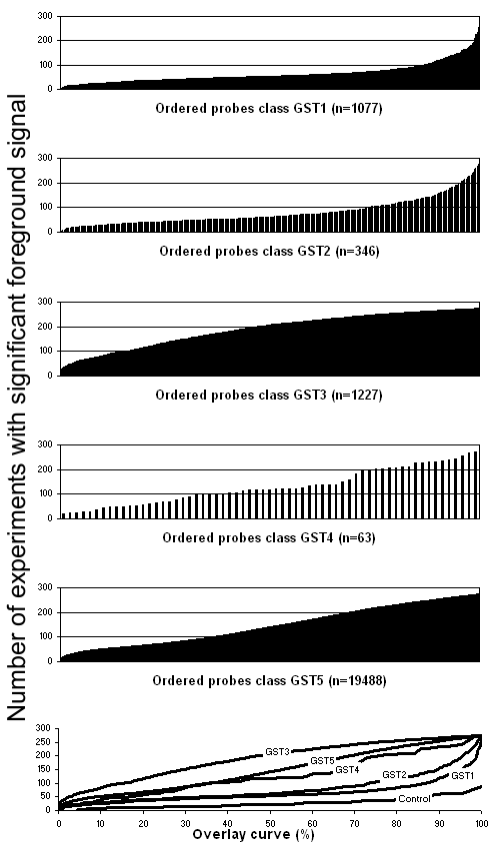
**Distribution of microarray foreground signal values according to GST classes**. In the five top panels, the graphs represent the number of microarrays experiments (out of a total of 276 hybridizations) in which a given probe had a statistically significant foreground signal. One bar corresponded to one GST feature that was ordered from left to right according to that number. The size (n) of each class is indicated in the top left corner of the five top graphs. The microarray design used for all experiments was registered as A-MEXP-58 in the ArrayExpress repository [17,18]. The graph in the bottom panel presents the curve overlay after scaling the X axis to 100% of the probes in each class. This graph also shows a cumulative distribution of the Cy3 channel signal of the Lucidea [21] background control spikes present on the microarray.

To test this hypothesis, we analyzed the results of 276 CATMA microarray experiments performed in the context of the European FP5 Compendium of Arabidopsis Gene Expression (CAGE) project [16]. The corresponding biological samples consist of various Arabidopsis anatomical parts, harvested at different development stages, after different treatments or stresses, and from mutants (Additional File 5), including links to ArrayExpress experiment accessions [17,18]. Figure 3 shows the number of experiments in which a GST feature (a microarray probe) was associated with statistically significant foreground signal values (see Methods) in the 276 microarray data sets. The five top graphs represent the experimentally observed hybridization characteristics for each of the five GST classes.

As expected, the median number of significant foreground value experiments per probe for each class could be ranked in the following order: GST1 (54; 19.6% of the total number of experiments) < GST2 (61; 22.1%) < GST5 (141; 51.1%) < GST3 (207; 75.0%). It is important to note that class size varied significantly. The GST4 curve was irregular and difficult to rank because it was constructed with data collected from very few features. Remarkably, GST1 and GST2 medians were far from negligible. This observation could be explained in different ways: (1) the hybridization conditions might yield unspecific signals; (2) GST1 and GST2 tags might still yield residual signal because of partial overlap with actual transcription units; (3) regions currently defined as intergenic, but nevertheless transcribed, might hybridize to GST1 or GST2 tags [19,20]. We favor the latter two explanations because the Cy3 hybridization signal pattern observed for the Lucidea [21] background control spikes printed on the CATMA array had a very low median number of experiments with a significant foreground signal (21; 7.6%), distinctly below the median number for probe classes GST1 and GST2. To summarize, our observations confirm that the distribution of hybridization signal values is in agreement with the GST classification established on the basis of DNA sequence analysis and that this analysis correctly predicts the quality of the GST features in microarray experiments.

### Design of the CATMAv4 addition including Gene Family Tags

In a final effort to reach comprehensive genome coverage of the CATMA repertoire, we had to resort to alternative strategies to design a tag for the remaining 2,338 untouched Arabidopsis nucleus-encoded TAIR6 cDNA sequences. Many of these genes lacked a matching GST because they belonged to gene families with high sequence similarity (above the 70% threshold previously chosen for GST design). To establish their relationship, the untouched genes were compared with each other and with the CATMAv3-tagged genes with NCBI-BLASTn [22] and grouped in gene families. The gene families were delimited to minimize the number of CATMAv3-tagged members they contained and the number of genes adhering to multiple families. For each family, a representative sequence was defined as a fragment from one of the members of the family, sharing at least 70% identity with all other untouched members and containing at least 50% of exon sequence. Using the Primer3 software [23], we designed sequence tags based on the representative family sequences. Tags corresponding to multi-member gene families were referred to as Gene Family Tags (GFTs), whereas tags corresponding to singleton genes were referred to as additional GSTs. An example of sequence alignment leading to the design of a GFT is presented in Additional File 6.

**Figure 4:**
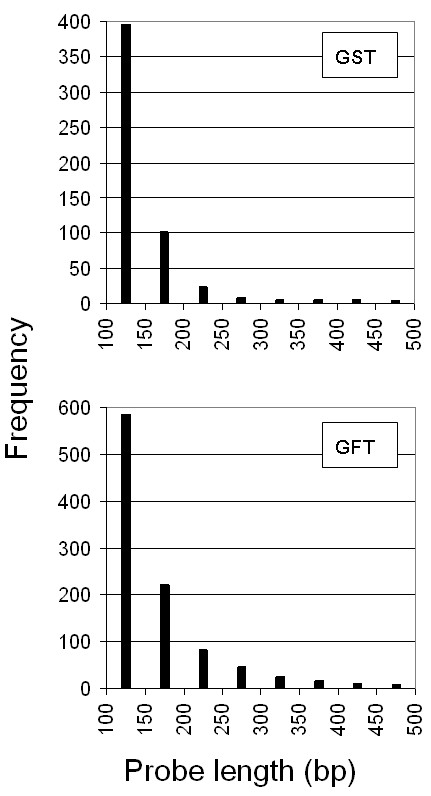
**Distribution of probe length for both GSTs and GFTs**. The distribution of the probe length is shown for all CATMAv4 addition features. The height of each bar corresponds to the number of probes in the length bins. Upper and lower panels represent the length distribution of the GFTs and GSTs, respectively.

**Figure 5:**
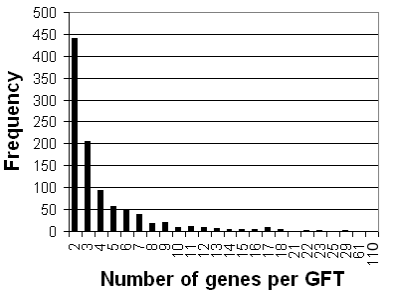
**Size of gene families**. The size of the gene families, reflecting the numbers of genes tagged by a single GFT, is determined by matching the GFT against the TAIR6 cDNA database with BLAST and subsequently aligning the BLAST hits against the genome. A BLAST hit target was included in the tagged gene family when its percentage sequence identity was higher than 70% and when the alignment confirmed an exon percentage higher than 50%. Corresponding gene family sizes are shown for all 990 designed GFTs.

To increase the chance of identifying GFTs or GSTs, the minimum amplicon length was lowered from 150 to 100 bp. Such tags still yield satisfactory hybridization signals (data not shown) [24], whereas shorter tags are difficult to purify from a PCR mix. As shown in Figure 4, the length of most newly designed tags was between 100 and 150 bp, justifying the choice to lower the length threshold as it greatly improved the ability for the tag design algorithm to identify appropriate sequences. Other criteria whose relaxation markedly affected the design included extension of primer GC content range to 20% to 95% (from 30% to 80%) and restricting the specificity calculation only to cDNA sequences. In all 2,338 genes, only three genes (At2g03937, At3g50250 and At4g19270) did not yield a satisfactory tag because of an unusually high proportion of GC or AT repeats. All additional 990 GFTs and 543 GSTs are listed in Additional File 7 and constitute the CATMAv4 addition. This information is also available online [14,25]. In addition, we assessed the specificity of the CATMAv4 GSTs, essentially as described above for the CATMAv2 and CATMAv3 probes. For 31% of these GSTs, a flag was added in the CATMA database, warning for potential off-target hybridization. A first amplification round for a subset of the CATMAv4 repertoire addition had an amplification success rate of 95% (data not shown). Note that 65% of the GFTs tagged only two or three genes (Figure 5), so that a positive hybridization result corresponding to a given feature in practice could easily be assigned to the responsible gene(s) in a follow-up study.

## Conclusion

We launched a significant effort to extend the existing CATMAv2 repertoire with additional GSTs to reach comprehensive genome coverage. The CATMAv2 GST repertoire was synchronized with two recent Arabidopsis genome annotations to identify 'orphan' genes. In two complementary design rounds, we could increase the number of sequence tags to 31,876. First, SPADS adjustments significantly increased the success of GST design at the cost of only a limited lower specificity. Subsequently, a sequence tag for nearly all remaining genes was selected by applying a markedly different design algorithm in which key design criteria were relaxed and single sequences allowed tagging of multiple genes of the same gene family. Counting all the genes satisfactorily tagged by a CATMAv2 GST (GE5) and the additional genes mapped by a v3 or v4 tag, the CATMA repository addresses 26,173 nuclear protein-coding TAIR6 annotated genes. CATMAv2 GSTs were carefully classified to predict and test their performance in microarray experiments. The analysis of a large set of microarray experiments validated both the correctness of this classification as well as the robustness of the CATMA microarrays. Interestingly, the analysis also revealed a substantial number of GSTs that now map to intergenic regions, but in hybridizations appear to be capable to detect transcripts in microarray experiments. As these GSTs were once designed to target annotated gene models, these gene models might have been incorrectly declared obsolete during subsequent genomic re-annotations.

This work concludes a unique voluntary collaboration of European laboratories funded by national government agencies to generate a valuable community resource that would not have been possible to create on a national basis.

## Methods

### Gene and GST classification

The TIGR5 (January 2004, [8]) and TAIR6 (November 2005, [13]) annotations were downloaded from the TAIR website and all gene models (including the splice variants) of the protein-encoding nuclear genes were extracted with a script based on the TIGR XML parser [26]. The EuGène Arabidopsis structural annotation was constructed in a three-step procedure. Firstly, the Arabidopsis nuclear genome sequence (Assembly TIGR5, [27]) was processed with the EuGène software (version 1.64 with SpliceMachine plugin [28] and ability to handle full pseudomolecule sequences). External information sources were Swissprot release 44, PIR release 79 and all EST and full-length cDNA sequences publicly available from the EMBL Nucleotide Sequence Database [29] on 17 September 2004. Secondly, the resulting coding sequences were re-mapped against EST libraries with NCBI-BLASTn [22], CAP3 [30] and SIM4 [31] to append UTRs to the gene models. Thirdly, Perl scripts removed two types of apparent annotation artifacts: small (<50 bp) outer exons, that were part of extended UTRs and distant by more than 2,000 bp from the main transcription unit, and unspliced UTRs longer than 3,200 bp. These cut-off values were deduced from distribution analysis of UTR, intron and exon size from gene models supported by cDNAs.

All CATMAv2 GST sequences were extracted from the CATMA database [14]. The CATMAv2 repertoire was compared to the nuclear TIGR5 genome sequence using NCBI-BLASTn (q = 1; e = 500; W = 7; filter false). GST BLAST hits and exon information were stored in a MySQL database. For both genome annotations, gene models without a predicted 3' UTR were extended beyond their stop codon with an additional, contiguous 150 bp to create an artificial 3' UTR. The length of 150 bp is a conservative figure below the average annotated 3' UTR length (227.5 bases). For 72% and 88% of the gene models including a 3' UTR, this region is >150 and >100 bases, respectively. Therefore, in the vast majority of cases, the addition of an artificial 3' UTR of 150 bp to predicted gene models lacking transcript support is unlikely to yield misplaced GSTs. Additionally, exons overlapping with exons of other genes were flagged and removed or, where possible, replaced by new 'partial exons' containing only non-overlapping sequences. Perl scripts were used to compare the coordinates of GSTs and gene models, and to sort genes and GSTs. Classification was first performed according to the TIGR5 structural annotation and then complemented with the 'unique' EuGène models. A EuGène model was defined as unique when no exon of a TIGR5 protein-encoding gene could be located between its 5' and 3' ends (both polarities).

### GST design for the CATMAv3 addition

As described in more detail elsewhere [3], SPADS first scans (predicted) transcribed sequences from 3' to 5' to identify with BLASTn [22] those regions with low similarity compared to the rest of the genome. Within these relatively unique regions, the software finds the best primer pairs for the PCR amplification of a gene-specific tag. The selection criteria are gradually relaxed when no primer pairs could be selected in the most divergent regions. When necessary, the design also allows GSTs to span introns. SPADS also calls on Primer3 [23] to select appropriate primer pairs and BLASTn to guarantee their specificity with regard to the amplification template. SPADS parameters used for GST design were as follows: GSTs should be 150 to 500 bp in length, with a maximum of 70% sequence identity (through its entire length) with any other Arabidopsis nuclear genome sequences, and possibly containing intron regions (at most 50% of intron sequence relative to total GST length in tags containing a minimum of 150-bp exon sequence). SPADS design parameters for PCR amplification primers included: length, 18 to 25 nucleotides; Tm, 50°C to 65°C (ΔTm<5°C); and GC%, 30% to 80%.

Only probes in the GST5 class were taken into account to calculate the quality characteristics of the CATMAv2 repository presented in Figure 3, but not when both probe ends were not positioned within an exon of the cognate gene. The relative position of a GST with regard to its cognate transcript sequence was determined as follows: when a GST started in the first third of the transcript and ended before the last third, it was assigned 5'; when the GST started after the first third and ended in the last third it was declared 3'; in all other cases, the GST was declared to be in central position.

### Microarray experiments

All spotted microarrays were hybridized with two samples simultaneously: the Cy5-labeled Arabidopsis cDNA and the 'universal reference' consisting of the 16 Cy3-labeled oligonucleotides complementary to the universal GST extensions added to the sequences with the PCR amplification primers (denoted as rA-rP in [5]). In this configuration, the reference label provided stable signal for all properly printed features. A gene was deemed transcribed (present call) when both the biological sample and the universal reference resulted in a significant foreground signal value (Fg) higher than the background signal value (Bg) plus twice the local standard deviation of the background signal (Fg > Bg + 2 σ(Bg)). All TAIR6 and EuGène 040917 gene models were considered to classify the GST features. By definition GST5 features uniquely tagged a gene in at least one of the structural annotations and GST1 features did not correspond to any gene in either annotation. Additional information about the microarray experiments is provided in Additional File 5 and available on line via ArrayExpress [18].

### GST and GFT design for the CATMAv4 repertoire addition

All scripts for designing the CATMAv4 repertoire addition were written in BioPerl. A flowchart of the CATMAv4 repertoire addition is provided in Additional File 8. The design process was started with cDNA sequences from the TAIR6 annotation. To produce a list of 'orphan' gene models, all AGI gene models were removed that were in any way tagged by a CATMAv3 repertoire GST, leaving 3,352 untouched genes classified as GE1 or GE2. We removed another 1,014 genes from this collection because they were smaller than 150 bp or were mitochondrial, chloroplastic or non-coding genes.

The coding sequences of the remaining 2,338 untouched genes were compared with each other with NCBI-BLASTn [22] to group them into families. A segment was selected in each gene where the number of co-aligned homologous sequences was the lowest. This 'representative family sequence' (RFS) served as reference for the corresponding gene family defined as all genes sharing regions with more than 70% sequence identity with the RFS. An RFS had to be longer than 150 bp and was preferentially positioned toward the 3' end of a gene.

The RFS was compared to the TAIR6 cDNA database with BLAST to assess any sequence similarity with previously tagged genes. RFS ends sharing more than 70% identity with such genes were trimmed down to a minimum length of 150 bp. Any CATMAv3-tagged genes still mapping in these trimmed RFS were included into the cognate gene family for subsequent analyses. Because a gene family was initially constructed for each gene, the resulting groups vastly overlapped. Redundant gene families were removed when all of their members were already represented either in other gene families with fewer members or in other gene families with fewer aggregated CATMAv3-tagged genes. With a Smith and Waterman algorithm [32] with a gap open penalty of 50 and a gap extension penalty of 10, each RFS was aligned to the genomic sequence of the family members.

Tag design was attempted for each RFS with Primer3 in two successive cycles. First, the primer GC content was constrained between 30% and 70%, the amplicon length between 120 and 500 bp, and the primer Tm had to be between 50°C and 65°C (ΔTm ≤ 5°C). When no satisfactory amplification primers could be identified, the second cycle parameters were GC content between 20% and 95% and minimum amplicon length down to 100 bp. For each successfully designed amplicon, a Smith and Waterman analysis verified whether the identity percentage over the entire amplicon was still exceeding 70% for each family member; if not, a new amplicon design was attempted. If no amplicon tagging the entire gene family could be selected, previously removed redundant gene families were recruited back to tackle 'orphan' family members.

## Note added in proof

The latest TAIR7 [33] Arabidopsis genome annotation was released after submission of this manuscript. All CATMAv4 GSTs (excluding GFTs) were classified and mapped onto the updated gene models as listed in Additional File 9 and available on line via the CATMA database [14]. According to the TAIR7 annotation, a few GSTs from the CATMAv2 repertoire (22) now tag a gene model. As expected, on average, the foreground signal for these GSTs was statistically significant in a higher number of microarray experiments in contrast to other GSTs that do not correspond to any TAIR7 gene model (Mann-Whitney U-test: p < 0.01)

## Abbreviations

AGI Arabidopsis Genome Initiative

BLAST Basic Local Alignment Search Tool

bp base pairs

CATMA Complete Arabidopsis Transcriptome MicroArray

EST Expressed Sequence Tag

EuGène Eukaryotic gene finder

GFT Gene Family Tag

GST Gene-specific Sequence Tag

NCBI National Center for Biotechnology Information

ORF Open Reading Frame

PCR Polymerase Chain Reaction

Perl Practical Extraction and Report Language

SPADS Specific Primer and Amplicon Design Software

TAIR The Arabidopsis Information Resource

TIGR The Institute for Genomic Research

UTR UnTranslated Region

## Competing interests

The author(s) declares that there are no competing interests.

## Authors' contributions

GS performed the GST design of the CATMAv3 repertoire addition and all related analyses and drafted the manuscript. RL and PR designed the CATMAv4 repertoire addition, carried out the corresponding analyses, and wrote and integrated the corresponding sections into the manuscript. JA carried out the CATMA microarray data analyses under supervision of YM. JB, RB, WN, JPR, PR, and PH amplified the GSTs of CATMAv3 addition by PCR, resulting in the actual physical upgrade of the CATMAv3 repertoire. BDM was responsible for maintaining, upgrading, and providing direct access to the CATMA database. MK and PH conceived the GST repertoire upgrade, assisted in making the major GST design decisions, and participated actively in the writing of the manuscript. All authors read and approved the final manuscript.

## Supplementary Material

Additional file 1 **EuGène 040917 Arabidopsis genome annotation.**. The text file lists the protein-encoding gene models identified in-house with the EuGène algorithm upon processing the reference chromosome molecules (ATH1_chr1.1con.01222004 to ATH1_chr5.1con.01222004, to be downloaded at [29]). The header of each gene model consists of the chromosome name, followed by the EuGène gene name (only used as internal reference). Each annotated gene name is followed by the list of its constituting exons, indicating exon type, start and stop chromosomal coordinates and strand polarity. Exon types include: coding sequence (cds), 5' UTR (5_utr), 3' UTR (3_utr) and extended UTR (e_utr) not flanking the translated sequence. The genes are listed according to their positional order on each successive chromosome. Click here for file

Additional file 2 **Flowchart of the gene classification algorithm.**. The graph illustrates the hierarchy of the decision criteria used in the gene classification algorithm. The colored arrows, white rectangles, and hatched rectangles represent gene exons, primary GST BLAST hits, and secondary GST BLAST hits, respectively. The bottom part of the figure contains the decision criteria (text in diamonds) and the subsequent classifications (text in colored boxes) or application of the next decision criterion. Click here for file

Additional file 3 **Mapping of CATMAv3 GSTs onto TAIR6 gene models**. Two different probe classifications are presented, according to the algorithm described in Additional File 2, for all GSTs of the v3 repertoire. The classification shown in column B only takes the TAIR6-annotated genes into account, whereas the classification shown in column C makes use both of all 040917 EuGène genes and TAIR6 genes. **Column A**: CATMA ID for each GST. **Column B**: GST class code (see Table 1) when only TAIR6 gene models are analyzed. All CATMAv3 GSTs were classified whether or not they were successfully amplified by PCR, while the gene classification at the basis of the CATMAv3 repertoire addition only included PCR-amplified GSTs. Note that a GST classified as GST3 does not necessarily tag more than one gene; in line with the classification definitions, this would be only the case if the overlap length with the non-target gene(s) were longer than 100 bp. **Column C**: GST class code when TAIR6 and EuGène 040917 gene models are analyzed collectively. A larger collection of possible target genes decreases the number of GST1 and GST2 calls. **Column D**: Comma-separated list of TAIR6 AGI code(s) of the nuclear protein-encoding gene model(s) tagged by GSTs of classes 3, 4 and 5 in column C. Taking into consideration that a small fraction of GSTs does not tag all the alternative splice forms of a certain gene, the name of the splice form is given instead of the gene name. **Column E**: Comma-separated list of EuGène 040917 ID(s) of the nuclear protein-encoding gene(s) tagged by GSTs of classes 3, 4 and 5 in column C. Click here for file

Additional file 4 **Detailed description of the CATMAv3 addition**. Information is also available on line at [14]. The listed features are: **Column A**: CATMA ID. **Column B**: Gene name of cognate gene according to the AGI (TIGR5) or EuGène annotation. **Column C**: GST length, excluding the universal GST extensions added within PCR primers. **Column D**: Amplification results. The letter 'G' refers to PCR amplification from genomic DNA template. The following numbers qualify the results of the PCRs as analyzed by DNA gel electrophoresis: 0, no detectable PCR product; 1, one product of the expected size; 2, multiple bands or DNA smear; 3, one product but with a wrong size. **Column E**: Fraction (%) of intron sequence compared to the total GST length (by design <50%); when the cognate gene had different splice variants (TIGR5), intron percentage was averaged over all splice variants. **Column F**: GC content; **Column G**: GST specificity expressed as percentage of sequence identity in the best non-trivial BLAST hit, i.e. not matching the cognate gene sequence, using the genome sequence as a BLAST database (by design <70%); **Column H**: 5' primer sequence, excluding the universal extension; **Column I**: 3' primer sequence, excluding the universal extension; **Column J**: Amplicon sequence, excluding the flanking universal extensions. Click here for file

Additional file 5 **CAGE microarray experiment overview**. The names of all CAGE microarray experiments used for the experimental verification of the GST classification are provided in a spreadsheet. **Column A**: CAGE experiment ID; **Column B**: ArrayExpress experiment accession number [18]; **Column C**: Analyzed Arabidopsis ecotype; **Column D**: Experimental factor under study; **Column E**: Growth medium; **Column F**: Number of individual hybridizations of the microarray experiment used for GST classification verification, i.e. those performed on the A-MEXP-58 microarray version; **Column G**: Examined plant anatomy part; **Column H**: List of different Boyes stages examined within the hybridizations referred to in column F. Click here for file

Additional file 6 **Structure and design of the gene family tag CATMA_GFT01324**. Genes are represented as boxes, identified by their AGI codes (TAIR6), and aligned with respect to their BLAST hit. For each gene, the hatched zone of the box represent regions where the sequence identity with the first gene (At3g48320, shown on top) is higher than 70%. Previously designed CATMAv3 GSTs are indicated with grey lines, together with their CATMA ID. The representative family sequence (RFS) is located in At3g48320 in the region where the number of highly homologous sequences is the lowest (delimited by vertical dotted lines). Amplicons were designed by Primer3 software in the RFS. Click here for file

Additional file 7 **Detailed description of the GFT and short GST sets**. Information is also available on line ([14,25]). **Column A**: CATMA ID; forming the fourth addition to the CATMA repertoire, the short GSTs have the letter 'd' in their ID. To emphasize the different underlying design paradigm, the GFTs have no specific letter, but instead 'GFT' in their ID. The numbering of both short GST and GFT IDs is random. A pseudomolecule number precedes the letter 'd' for the short GSTs. This information is not included in the ID of GFTs, because they can correspond to genes residing on different pseudomolecules. **Column B**: AGI code of cognate gene according to TAIR6 annotation; **Column C**: Other family members listed by AGI gene name (only for GFTs); **Column D**: GST length, excluding the universal GST extensions added with PCR primers; **Column E**: Fraction of intron sequence compared to the total GST length when the cognate gene had different splice variants (TAIR6) intron percentage was averaged over all splice variants. **Column F**: GC content; **Column G**: GST specificity expressed as percentage of sequence identity in the next best BLAST hit, using the genome sequence as a BLAST database; this parameter was only calculated for the GSTs; the parameter is not a meaningful quality criterion for the GFTs because they are designed to bear a high sequence similarity with the gene family members. Note that some of the values are larger than 70% as a consequence of the use of a different scope (cDNA) for specificity verification during the design of the CATMAv4 probes. **Column H**: 5' primer sequence, excluding the universal extension; **Column I**: 3' primer sequence, excluding the universal extension; **Column J**: Amplicon sequence, excluding the flanking universal extensions. Click here for file

Additional file 8 **Flowchart of the CATMAv4 repository addition design**. The flowchart illustrates the order of analysis and filtering steps and gene numbers leading to the clustering of genes into gene families, the selection of corresponding representative family sequences, and the design of GSTs or GFTs using the representative family sequences as template. The actual design process is described in Methods. Click here for file

Additional file 9 **Mapping of CATMAv4 GSTs onto TAIR7 gene models**. The classification shown in column B only takes the TAIR7-annotated genes into account, whereas the classification shown in column C takes both EuGène 040917 and TAIR7 genes. **Column A**: CATMA ID for each GST. **Column B**: GST class code (see Table 1) when only TAIR7 gene models are analyzed. Classification was performed for all CATMAv4 GSTs whether or not they were successfully amplified by PCR. **Column C**: GST class code when TAIR7 and EuGène 040917 gene models are analyzed collectively. **Column D**: Comma-separated list of TAIR7 AGI code(s) of the nuclear protein-encoding gene model(s) tagged by GSTs of classes 3, 4 and 5 in column C. Taking into consideration that a small fraction of GSTs does not tag all the alternative splice forms of a certain gene, the name of the splice form is given instead of the gene name. **Column E**: List of textual descriptions of the genes listed in column D. One description is given per gene, not distinguishing between different splice variants. In case of multiple genes listed in column D, the different gene descriptions are separated by a '@' character. The descriptions correspond to a concatenation of the 'COM_NAME' and 'PUB_COMMENT' fields from the TAIR7 annotation files [33]. **Column F**: Comma-separated list of EuGène 040917 ID(s) of the nuclear protein-encoding gene(s) tagged by GSTs of classes 3, 4 and 5 in column C. Click here for file
