# Supplementary figures and images for: CATMA, a comprehensive genome-scale resource for silencing and transcript profiling of Arabidopsis genes
Source: BMC Bioinformatics. 2007 Oct 18;8:400. doi: 10.1186/1471-2105-8-400 (PMC2147040; doi:10.1186/1471-2105-8-400)

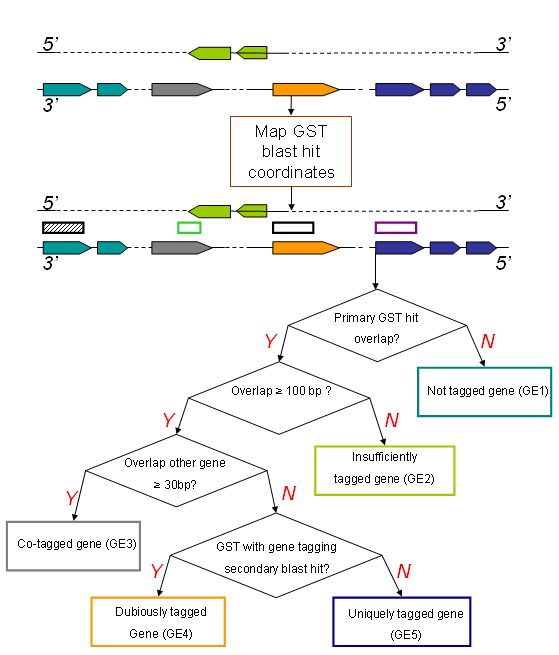

Supplement: Additional file 2 — Flowchart of the gene classification algorithm.. The graph illustrates the hierarchy of the decision criteria used in the gene classification algorithm. The colored arrows, white rectangles, and hatched rectangles represent gene exons, primary GST BLAST hits, and secondary GST BLAST hits, respectively. The bottom part of the figure contains the decision criteria (text in diamonds) and the subsequent classifications (text in colored boxes) or application of the next decision criterion. [file 1471-2105-8-400-S2.png]

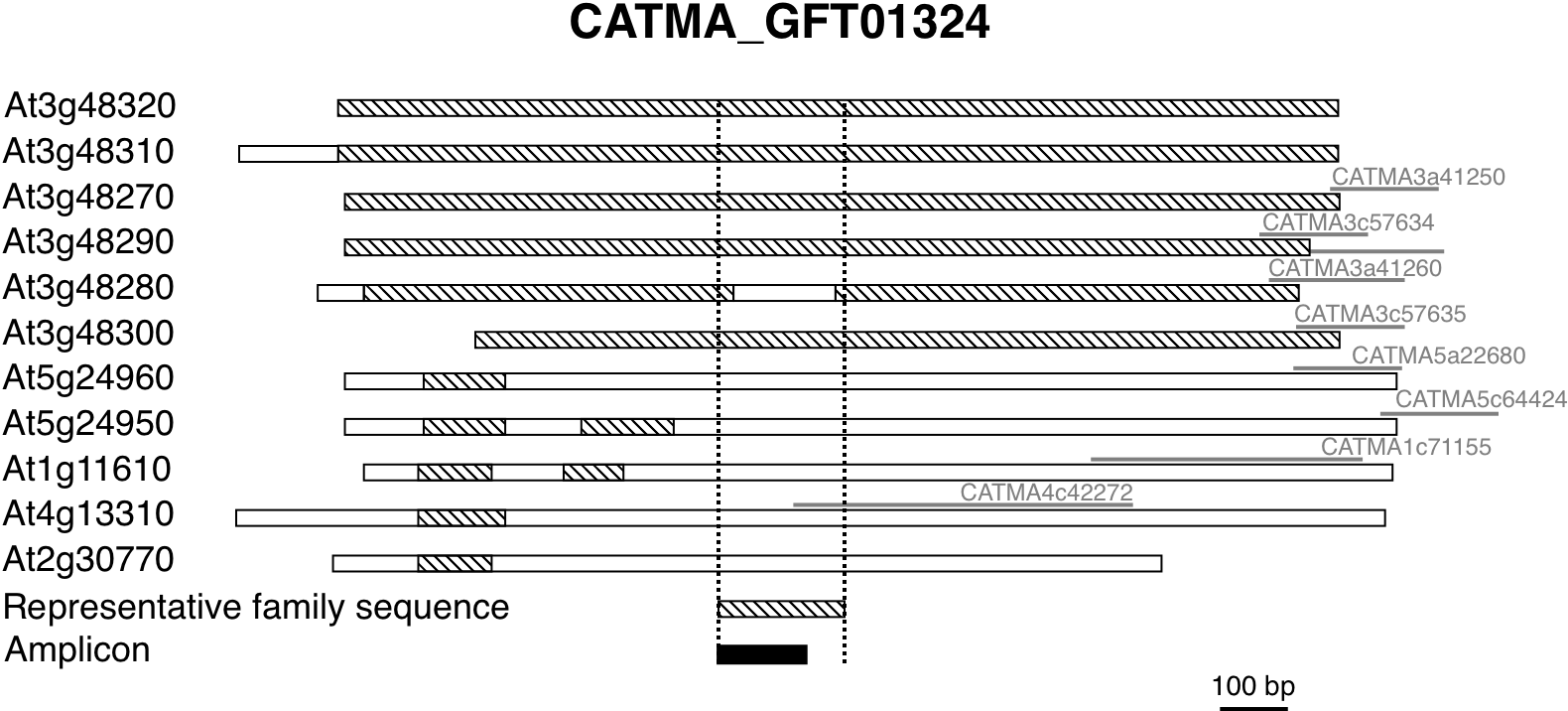

Supplement: Additional file 6 — Structure and design of the gene family tag CATMA_GFT01324. Genes are represented as boxes, identified by their AGI codes (TAIR6), and aligned with respect to their BLAST hit. For each gene, the hatched zone of the box represent regions where the sequence identity with the first gene (At3g48320, shown on top) is higher than 70%. Previously designed CATMAv3 GSTs are indicated with grey lines, together with their CATMA ID. The representative family sequence (RFS) is located in At3g48320 in the region where the number of highly homologous sequences is the lowest (delimited by vertical dotted lines). Amplicons were designed by Primer3 software in the RFS. [file 1471-2105-8-400-S6.png]

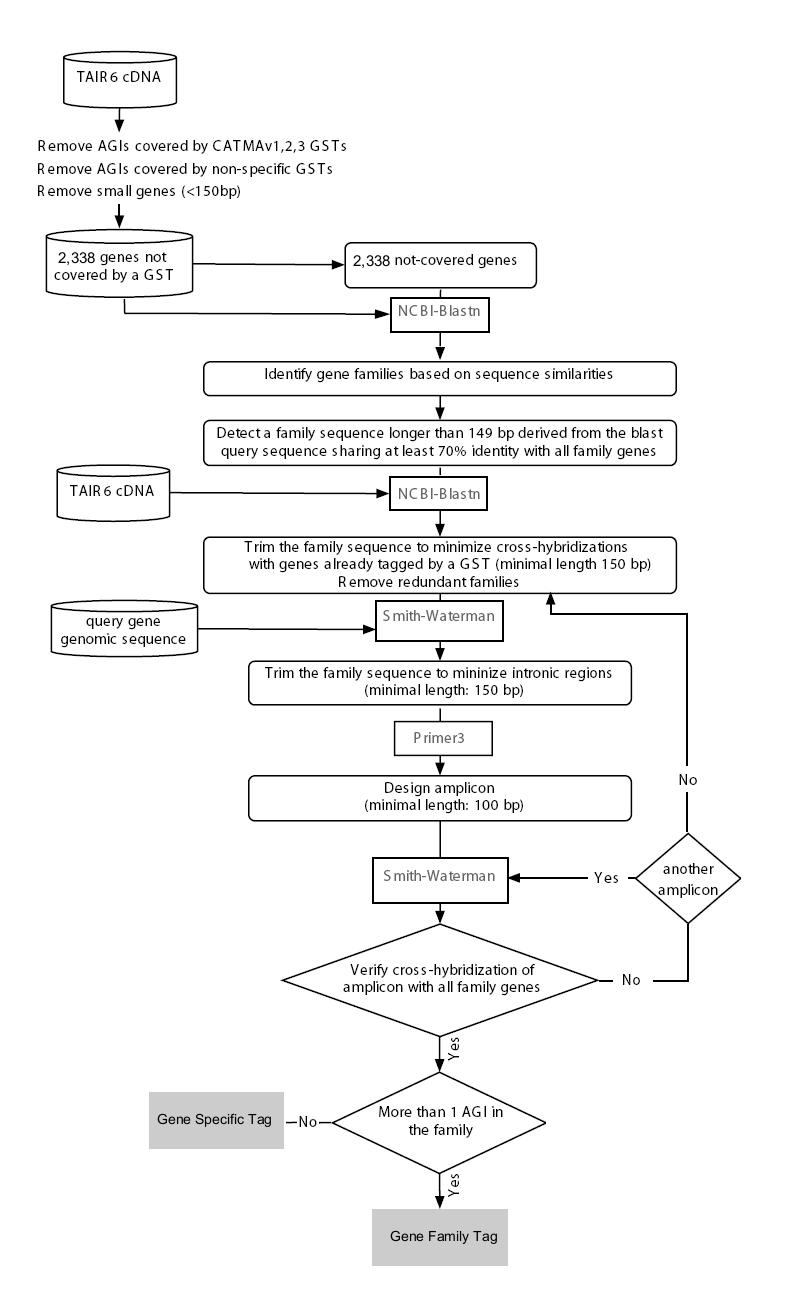

Supplement: Additional file 8 — Flowchart of the CATMAv4 repository addition design. The flowchart illustrates the order of analysis and filtering steps and gene numbers leading to the clustering of genes into gene families, the selection of corresponding representative family sequences, and the design of GSTs or GFTs using the representative family sequences as template. The actual design process is described in Methods. [file 1471-2105-8-400-S8.png]
